# Sustainability of a well-established hand hygiene program during the coronavirus disease 2019 (COVID-19) pandemic

**DOI:** 10.1017/ash.2022.13

**Published:** 2022-03-02

**Authors:** Lisa B. Stancill, Emily E. Sickbert-Bennett, Lauren M. DiBiase

**Affiliations:** 1 Department of Infection Prevention, University of North Carolina at Chapel Hill Medical Center, Chapel Hill, North Carolina; 2 University of North Carolina Gillings School of Global Public Health, Chapel Hill, North Carolina; 3 UNC School of Medicine, University of North Carolina, Chapel Hill, North Carolina

**Keywords:** Hand hygiene, COVID-19

## Abstract

Overall, engagement and compliance from the crowd-sourced hand hygiene observation program, Clean-In-Clean-Out (CICO), were similar between 2019 (96.6%) and 2020 (96.7%) despite fluctuations within 2020 that reflected our hospital’s coronavirus disease 2019 (COVID-19) experience. Shared responsibility and just-in-time reminders can allow manual hand hygiene observation models to be sustainable.

Hand hygiene is a cornerstone of infection prevention and is routinely tracked in hospital settings. During the coronavirus disease 2019 (COVID-19) pandemic, the main focus has been on airborne and droplet transmission, but the importance of hand hygiene has also had renewed emphasis. Severe acute respiratory coronavirus virus 2 (SARS-CoV-2) can survive on human skin for ∼9 hours, but it is easily inactivated with an alcohol-based hand rub, reinforcing the role of hand hygiene in limiting the COVID-19 spread.^1^

To date, evaluations of hand hygiene in hospital settings during COVID-19 have focused on automated monitoring systems which collect compliance data through badge readers and/or dispenser use, eliminating the need for staff to perform traditional hand hygiene observations.^2–4^ By contrast, we sought to determine whether UNC Medical Center’s Clean-In-Clean-Out (CICO) program,^5^ a well-established albeit manual method for hand hygiene observations, was sustainable throughout a public health and healthcare crisis. We also sought to determine whether the COVID-19 pandemic had an effect on hand hygiene compliance by longitudinally examining key metrics of the CICO program.

## Methods

UNC Medical Center utilizes a crowd-sourced hand hygiene audit application, CICO, to track hand hygiene observations and compliance.^5^ Briefly, CICO can be accessed on any device on the hospital network via a web browser in all care settings. All staff are invited to participate with a suggested 5 observations per month, and they are encouraged to provide real-time feedback (compliment or reminder) when performing observations.^5,6^

During this evaluation, key hand hygiene metric data were queried from the CICO application regarding the following elements: (1) engagement, the absolute number of observations performed overall and per 1,000 patient days; (2) compliance, hand hygiene compliance percentage; and (3) accountability, the percentage of observations with feedback from January 2019–December 2020 across inpatient settings. Weekly counts of COVID-19 hospitalized patients were determined by the weekly average number of COVID-19 patients on the midnight census.

We made several comparisons to assess changes in data across 3 distinct periods. (1) Calendar year 2019 (baseline) was compared to calendar year 2020. (2) Calendar year 2020 was stratified into 4 periods based on the changing COVID-19 experience at our facility as follows: January–February was considered a prepandemic phase, with no COVID-19 patients, high census, and heightened awareness of COVID-19; March–June was characterized by COVID-19 patients and decreased overall census; July–September experienced the first COVID-19 surge with a return to high census; October–December was marked by high, sustained COVID-19 admissions along with high overall census. (3) To assess seasonal variation in hand hygiene compliance, October–December 2020 was compared to that from October–December 2019.

The 95% confidence intervals (CIs) were calculated using the standard formula for 95% CI for each of the periods and were compared to the 2019 baseline.^7^ Similarities were assessed by overlapping confidence intervals. Results are reported as mean with 95% CI.

## Results

### Engagement metric: observations

Overall, there were 97,429 observations in 2019 and 74,809 in 2020. Adjusting for the number of observations per 1,000 patient days showed no statistically significant difference between 2019 (318.4; 95% CI, 276.7–360.1) and 2020 (258.1; 95% CI, 229.1–287.1). Notably, total observations decreased below baseline levels during both the July–September (221.0; 95% CI, 203.0–239.0) and October–December periods (206.2; 95% CI, 190.1–222.4).

**Fig. 1.**
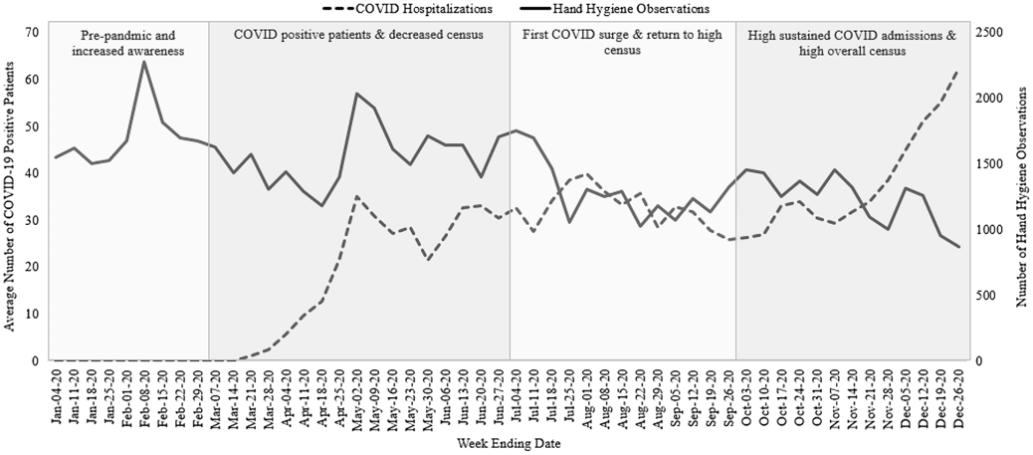
Count of inpatient hand hygiene observations each week along with average COVID-19 patient census since UNC Medical Center began seeing patients with COVID-19 in March–December 2020.

Hand hygiene observations followed the same trend (sharp increase followed by stabilization) as COVID-19 hospitalizations from March through July 2020, when the number of COVID-19 hospitalizations reached its first peak (Fig. 1). After mid-July, the number of observations stabilized and began decreasing with increasing hospitalizations. Hand hygiene observations never recovered to the spring 2020 level.

### Accountability metric: feedback

**Fig. 2.**
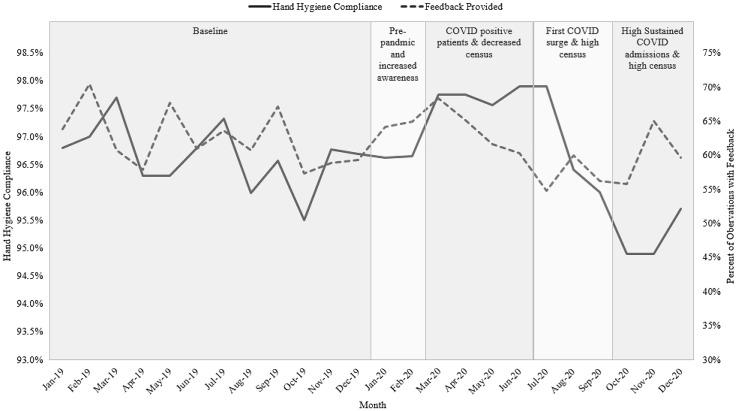
Inpatient hand hygiene compliance and percentage of observations with feedback provided for UNC Medical Center measured through the Clean-In-Clean-Out hand hygiene program from 2019 to 2020.

Although observations with feedback fluctuated month to month (Fig. 2), the feedback for 2020 (61.3%, 59.0-63.6%) was similar to feedback during all of 2019 (62.4%; 95% CI, 60.1%–64.6%). In October–December 2019, feedback was at the lowest yearly level, but it increased each month during the first quarter of 2020. However, this increase was not significantly different than the October–December 2019 baseline period (64.6%; 95% CI, 64.0%–65.1%). The percentage of observations with feedback only significantly decreased beyond baseline during one period, July–September 2020 (57.0%; 95% CI, 54.5%–59.5%).

### Compliance metric: compliance

Hand hygiene compliance as assessed by CICO was statistically similar in 2019 (96.6%; 95% CI, 96.3%–97.0%) and 2020 (96.7%; 95% CI, 96.0%–97.3%) (Fig. 2). However, across 2020 there was a statistically significant increase during March–June 2020 (97.7%; 95% CI, 97.6%–97.9%). However, compliance during October–December 2020 (95.1%; 95% CI, 94.7%–95.6%) decreased from the 2019 baseline and compared to October–December 2019 (96.3%; 95% CI, 95.7%–97.0%).

## Discussion

Not only was the Clean-In-Clean-Out model sustainable through a public health and healthcare crisis, initial increases in engagement and compliance metrics were identified. Although not at statistically significant levels, observations and feedback increased with COVID-19 media attention, prior to cases occurring in North Carolina or at our facility. These increases may be a reflection of staff proactively engaged in COVID-19 infection prevention efforts. Feedback and compliance increased again when our facility began seeing COVID-19 patients, even in the absence of active reminders and campaigns from infection prevention staff.

A statistically significant increase in hand hygiene compliance was observed during one period, March–June 2020, which corresponded with the admission of the first COVID-19 patient at our facility, shelter-in-place orders community-wide, and low inpatient volumes. In July, patient volumes started to return to pre–COVID-19 levels while our facility also began treating a higher number of COVID-19 patients. In addition, the hospital started experiencing staff shortages. The decrease in engagement observed with higher patient volume and patient-to-staff ratios is further evidence that operating at high capacity is not beneficial for patient safety.^8–10^ This trend was further demonstrated by the decrease in hand hygiene compliance from October through December 2020, when our facility had high, sustained COVID-19 admissions as well as a normal non–COVID-19 patient volume.

This study had several limitations. We evaluated hand hygiene at a single, large academic medical center, and these results may not be generalizable to other settings or hand hygiene programs. However, the results were similar to findings published on COVID-19 and automated hand-hygiene monitoring systems in which compliance increased with lower patient volume, especially early in the pandemic.^2,3^ CICO is a novel hand hygiene program and is subject to selection and measurement bias. However, CICO provides benefits of real-time feedback that directly affects practices.^5,6^

Hand hygiene compliance remained high, >90%. Key CICO metrics demonstrated that although a manual rather than automated monitoring tool requires extra effort from staff, it is still a viable model. Unlike automated systems, CICO provides the opportunity for just-in-time reminders. Stangerup et al^4^ found that hand hygiene compliance decreased when hand-hygiene data sharing stopped. Furthermore, the crowd-sourced component of CICO allows all staff to complete observations and provide real-time feedback as time permits rather than further burdening infection prevention staff or specific designees who were inundated with competing priorities.

The COVID-19 pandemic has caused healthcare staff to juggle numerous responsibilities with quality improvement and patient safety projects often being deprioritized and/or delayed. In the midst of this environment, it was encouraging to see that this CICO manual hand hygiene program was still actively utilized at comparable levels to the pre-COVID-19 baseline and that overall compliance and feedback provided did not significantly decline. In addition, the sustainability and success of the CICO program allowed UNC Medical Center to replicate this model of shared responsibility for monitoring mask compliance in contrast to more labor-intensive methods.^11^ In the midst of a global pandemic, sustainable hand hygiene programs remain foundational to the success of infection prevention.
